# 20-HETE Enzymes and Receptors in the Neurovascular Unit: Implications in Cerebrovascular Disease

**DOI:** 10.3389/fneur.2020.00983

**Published:** 2020-09-04

**Authors:** Ezekiel Gonzalez-Fernandez, Daniel Staursky, Kathryn Lucas, Bond V. Nguyen, Man Li, Yedan Liu, Chad Washington, Lique M. Coolen, Fan Fan, Richard J. Roman

**Affiliations:** ^1^Department of Pharmacology and Toxicology, University of Mississippi Medical Center, Jackson, MS, United States; ^2^William and Carey University College of Osteopathic Medicine, Hattiesburg, MS, United States; ^3^Department of Neurosurgery, University of Mississippi Medical Center, Jackson, MS, United States; ^4^Department of Biological Sciences, Kent State University, Kent, OH, United States

**Keywords:** 20-HETE, GPR75, neurovascular unit (NVU), blood brain barrier (BBB), cerebral blood flow (CBF), neurodegeneration

## Abstract

20-HETE is a potent vasoconstrictor that is implicated in the regulation of blood pressure, cerebral blood flow and neuronal death following ischemia. Numerous human genetic studies have shown that inactivating variants in the cytochrome P450 enzymes that produce 20-HETE are associated with hypertension, stroke and cerebrovascular disease. However, little is known about the expression and cellular distribution of the cytochrome P450A enzymes (CYP4A) that produce 20-HETE or the newly discovered 20-HETE receptor (GPR75) in the brain. The present study examined the cell types and regions in the rat forebrain that express CYP4A and GPR75. Brain tissue slices from Sprague Dawley (SD), Dahl Salt-Sensitive (SS) and CYP4A1 transgenic rat strains, as well as cultured human cerebral pericytes and cerebral vascular smooth muscle cells, were analyzed by fluorescent immunostaining. Tissue homogenates from these strains and cultured cells were examined by Western blot. In the cerebral vasculature, CYP4A and GPR75 were expressed in endothelial cells, vascular smooth muscle cells and the glial limiting membrane of pial arteries and penetrating arterioles but not in the endothelium of capillaries. CYP4A, but not GPR75, was expressed in astrocytes. CYP4A and GPR75 were both expressed in a subpopulation of pericytes on capillaries. The diameters of capillaries were significantly decreased at the sites of first and second-order pericytes that expressed CYP4A. Capillary diameters were unaffected at the sites of other pericytes that did not express CYP4A. These findings implicate 20-HETE as a paracrine mediator in various components of the neurovascular unit and are consistent with 20-HETE's emerging role in the regulation of cerebral blood flow, blood-brain barrier integrity, the pathogenesis of stroke and the vascular contributions to cognitive impairment and dementia. Moreover, this study highlights GPR75 as a potential therapeutic target for the treatment of these devastating conditions.

## Introduction

Stroke and dementia are leading causes of serious-long term disability and mortality in the United States (1, 2). These conditions share significant overlapping pathophysiology and associated risk factors (3). For example, hypertension is a well-known modifiable risk factor for both stroke and vascular dementia. The incidence of stroke doubles the chance of developing dementia (4). However, little is known about genes and pathways that determine susceptibility to stroke and dementia. Recently, inactivating variants in the cytochrome P450 enzymes (CYP4A11 and CYP4F2) that produce 20-hydroxyeicosatetraenoic acid (20-HETE) have been linked to both stroke, hypertension and cognitive impairment in numerous human genetic association studies (5–16).

20-HETE is a metabolite of arachidonic acid that is produced by CYP enzymes of the 4A11, 4F2, and 4F3 families in humans. *Cyp4A1, 4A2 4A3*, and *4A8* are the homologous isoforms that generate 20-HETE in rats. *Cyp4A1* is the most active isoform in rats (17, 18). 20-HETE is a vasoconstrictor that activates PKC, MAPK, tyrosine kinase and Rho kinase to promote Ca^2+^ entry through depolarization of vascular smooth muscle cells (VSMCs) secondary to blockade of the large-conductance Ca-sensitive potassium channel (19–21). Elevations in transmural pressure increase the production of 20-HETE, and inhibitors of this pathway decrease the myogenic response of renal and cerebral arteries and autoregulation of blood flow in both vascular beds (22–24). 20-HETE levels increase following subarachnoid hemorrhage (SAH) (25–27) and ischemic stroke (28, 29). Blockade of 20-HETE attenuates cerebral vasospasm following SA (25, 26), and infarct size following ischemia (30–32). Other studies have suggested that the local synthesis and release of 20-HETE by astrocytes or neurons may attenuate the dilation of penetrating arterioles in cerebral slices *in vitro* and functional hyperemic responses *in vivo* (33).

Recently, Garcia et al. identified a G_q_-coupled receptor (GPR75) that mediated response to 20-HETE in endothelial cells using click chemistry methodologies (34). The identification of a receptor for 20-HETE was a milestone in the eicosanoid field (35). GPR75 was previously suspected as an endogenous receptor for the chemokine RANTES/CCL5 (36). Activation of GPR75 by CCL5 was reported to attenuate the neurotoxic effect of amyloid-β, which is a hallmark of Alzheimer's disease (36). The effects of 20-HETE on this receptor in the brain remain to be determined.

While functional studies using 20-HETE inhibitors suggest a potential role for 20-HETE in the control of cerebral blood flow and cerebrovascular pathology, little is known about the cell types and regions of the brain that express the enzymes that produce 20-HETE or its newly discovered receptor, GPR75 (33, 37–40).

The 20-HETE pathway is poised to be a therapeutic target. 20-HETE antagonists, analogs, inducers and inhibitors have all been identified (41). Most are effective at nanomolar concentrations, highly lipid-soluble and capable of crossing the blood-brain barrier (BBB) (41). Identifying the cells types and regions in the brain that produce 20-HETE and express its receptor is a critical first step toward unraveling the complex mechanisms by which 20-HETE contributes to cerebrovascular disease and for the development of drugs to target this pathway.

## Methods

### Animals

Experiments were performed using (14–19 week old) Sprague Dawley (SD), Dahl Salt-Sensitive (SS) and CYP4A1 transgenic (SS.CYP4A1) rats obtained from colonies maintained at the University of Mississippi Medical Center. Generation of the SS.CYP4A1 rat was described in detail by Fan and coworkers (42). The rats were pair housed in barrier cages and had free access to food and water throughout the study. All animal protocols were approved by the Institutional Animal Care and Use Committee (IACUC) of the University of Mississippi Medical Center and were conducted in accordance with the NIH Guide for the Care and Use of Laboratory Animals (43).

### Tissue Collection and Sectioning

Animals were deeply anesthetized with 4% isoflurane. Brains collected for immunohistochemistry (IHC) were post fixed in 4% paraformaldehyde in 0.1 M sodium phosphate buffer (PB) for 24 h and then transferred to a 0.1 M PB preservation solution containing 20% sucrose and 0.01% sodium azide.

A subset of animals (*n* = 6 rats) were anesthetized with ketamine (30 mg/kg, i.m.) and Inactin (50 mg/kg, i.p.; T133, Sigma Chemical Corp, St. Louis, MO). Catheters were inserted into the femoral artery to record blood pressure and in the femoral vein for injection of a high molecular weight (500 kDa) FITC-Dextran (FITC-Dex) (10 mg/kg; FD500S, Thermo Fisher Scientific, Waltham, MA) that is impermeable to vessels in order to visualize the vasculature. FITC-Dex was allowed to circulate for 15 min prior to sacrifice. Mean arterial pressure for each animal was confirmed between 90 and 110 mmHg prior to tissue collection. The brains were then collected and post fixed in 4% paraformaldehyde and processed as described above.

The brains were sectioned coronally into 4 parallel series of 60 μm sections using a sliding microtome (H400R, Micron, Germany) equipped with a freezing stage (BFS-5, Physitemp, Clifton, New Jersey) or by a vibrating microtome (Compresstome VF-300-0Z, Precisionary Instruments, Greenville, NC). The sections were stored in a cryopreservative solution containing 30% sucrose (S7903, Sigma), 30% ethylene glycol (E178-1, Fisher), and 0.01% sodium azide (S227, Fisher) in 0.1 M PB, pH 7.35 at −20°C.

### Western Blot Analysis

Brains collected for Western blots were flash frozen after collection in liquid nitrogen and then homogenized in ice-cold radioimmunoprecipitation assay buffer (R0278, Sigma) with a protease inhibitor (A32959, Fisher). The homogenate was centrifuged at 9,000 g at 4°C for 15 min, and the supernatant was collected. Aliquots of the homogenates (20 μg protein) were resolved by electrophoresis on a 4–20% Criterion TGX gel (Biorad, Berkley, CA), transferred to a nitrocellulose membrane, and probed with a 1:1,000 dilution of two GPR75 antibodies which target different epitopes (ab75581, Abcam, Boston, MA) or (LS-A1589, LS-Bio, Seattle, WA) or a CYP4A1 antibody (RPAP-151, Cypex, Scotland, UK). The membranes were incubated with a 1:2,000 dilution of HRP coupled, goat anti-rabbit-IgG secondary antibody (ab6721, Abcam) for GPR75 or a 1:5,000 dilution of HRP coupled, donkey anti-goat-IgG secondary antibody for CYP4A1 (sc-2020, Santa Cruz, Dallas, TX). Membranes were re-probed for GAPDH as a loading control using a 1:5,000 dilution of a primary antibody (2118S, Cell Signaling, Technologies, Danver, MA) and a 1:10,000 dilution of the secondary antibody (ab6721). For comparison of the expression of GPR75 in other tissues, samples of rat liver and kidney were prepared and loaded on the same gels. HEK293 cell lysate (20 μg protein) was included as a negative control (LY416418, Origene, Rockville, MD) and run alongside brain homogenates as a positive control for GPR75 WB analysis. Liver microsomes (1 μg protein) prepared from a rat treated with fenofibrate for 3 days to induce CYP4A1 expression was used as positive control for CYP4A1 WB analysis. Brain tissue for WB analysis was obtained from 5 SD, 3 Dahl SS and 3 SS.CYP4A1 rats.

### Immunofluorescence

#### General

All immunostaining was performed using 60 μm free-floating coronal sections under gentle agitation. Primary incubations were performed at room temperature (RT) for 17 h for antibodies requiring the ABC-TSA (Avidin Biotin Complex -Tyramine Signal Amplification) method or for 4°C for 72 h for protocols using standard indirect immunofluorescence methods. Control and experimental sections from different groups of animals that were compared for quantification were immunostained simultaneously. Sections were first washed with phosphate-buffered saline (PBS; 0.1M sodium phosphate containing 0.9% sodium chloride) 12 times for 5 min prior to immunostaining. Sections that were immunostained using the ABC-TSA method were then treated with 1% H_2_0_2_ for 10 min. All sections were then pretreated for 1 h in the antibody incubation solution (PBS with 0.4% Triton X-100) containing 0.1% bovine serum albumin (J64655, Fisher). All primary and secondary antibodies were incubated in the presence of this antibody incubation solution. After immunostaining, the sections were washed with PB and then mounted on SuperFrost Plus glass slides (22037-246, Fisher) and coverslipped with antifade mounting medium (H-1400, VectaShield, Burlingame, CA) or (ProLong Gold with DAPI; P36931, Fisher). The sections were protected from light and stored at 4°C. Additional information regarding the CYP4A and GPR75 antibodies used in this study can be found in Table S1.

#### Imaging

Slides were imaged using a Nikon C2 laser scanning confocal system mounted on an Eclipse Ti2 inverted microscope (Nikon, Melville, NY) and processed using NIS Elements Imaging Software (4.6). All images for comparative expression studies were captured using identical laser, photomultiplier gain and exposure settings.

#### Identification of CYP4A in Neurons and Astrocytes

One series of parallel sections from the FITC-dextran perfused group of animals was chosen (n = 3 rats), washed in PBS, treated with H_2_0_2_ and blocked in the antibody incubation solution as described above. Sections were co-incubated in primary antibodies: CYP4A1 (RPAP-151; 1:1,000) and NeuN conjugated to Alexa Fluor 647 (ab190565, Abcam; 1:100) or GFAP (ab5804, Sigma; 1:200) for 17 h at RT followed by secondary incubations in biotinylated donkey anti-goat (A16003, Fisher; 1:500; 1 h), ABC-elite (PK-6100, Vector; 1:1,000 in PBS; 1 h), tyramine signal amplification (SAT700B001EA, Perkin Elmer, Waltham, MA; 1:250 in PBS plus 1 μL of 3% H202/mL PBS; 10 min) followed by Dylight 550-conjugated streptavidin (84542, Fisher; 1:200; 30 min) and Alexa Fluor 647 (ab150075, Abcam; 1:200; 30 min).

#### Quantification of CYP4A Expression in Neurons and Astrocytes

Tissue that had been immunostained for CYP4A1/NeuN as detailed above was examined and non-adjacent sections between Bregma −2.56 through −3.14 mm that encompassed the barrel cortex and hippocampus were selected. A 60x oil lens was used and a measurement frame of 50 x 50 μm was applied when counting neurons. A 20x lens was used and a 300 x 300 μm measurement frame was applied when counting astrocytes. A total of 5 measurement frames from the hippocampus were chosen that spanned the CA1-CA3 region and 5 frames at random were chosen in the somatosensory barrel cortex (S1BF) in 4 non-adjacent sections when counting neurons. A total of 3 measurement frames from the hippocampus were chosen when counting astrocytes. Neurons expressing both NeuN and CYP4A and astrocytes expressing both GFAP and CYP4A were counted. A typical confocal image of the hippocampus along with a zoomed in measurement frame is depicted in **Figure 7**. Three animals were analyzed in this manner. Neuron counts = (5 measurement frames per region × 4 non-adjacent sections × 3 animals), *n* = 60. Astrocyte counts = (3 measurement frames ×2 non-adjacent sections ×3 animals), *n* = 18.

#### Identification of GPR75 in Neurons and Astrocytes

Sections were co-incubated in primary antibodies: GPR75 (75581, Abcam; 1:4,000) and NeuN (ab190565, Abcam; 1:100) or GFAP (ab5804; 1:200) for 17 h followed by secondary incubations in biotinylated donkey anti-rabbit (A16039, Fisher; 1:500; 1 h), ABC-elite (PK-6100; 1:1,000 in PBS; 1 h), tyramine signal amplification (SAT700B001EA; 1:250 in PBS plus 1 μL of 3% H202/mL PBS; 10 min) followed by Dylight 550-conjugated streptavidin (84542; 1:200; 30 min). Two subsets of sections were also processed for comparison with additional GPR75 antibodies: (LS-A1589 & LS-A1594) at the same concentrations, times and temperatures listed above.

#### Regional Analysis of CYP4A and GPR75 Expression

Representative sections encompassing major anatomical landmarks between Bregma 0.2 and Bregma −5.30 were examined under a 4x magnification lens using standard fluorescent settings. Regions were examined for CYP4A or GPR75 cell bodies regardless of cell type and provided a density score of abundant, moderate, sparse or absent/nearly absent. Regions examined were correlated to the Waxholm Space atlas of the Sprague Dawley rat brain and Scalable Brain Atlas (44, 45).

#### Identification of CYP4A and GPR75 in Vascular Smooth Muscle Cells and Endothelial Cells

One series of parallel sections from 3 rats were co-incubated in CYP4A1 (RPAP-151; 1:200) or GPR75 (LSA1589; 1:200) and aSMA (anti-α-smooth muscle actin) (A2547, Sigma; 1:200) for 72 h at 4°C. Following primary incubations the sections were then incubated in Dylight 550 (Abcam; Cat# ab96932; 1:200 or AlexFluor 555 (A21428, Fisher; 1:200) for 30 min followed by AlexaFluor 488 (Fisher, Cat# A32766; 1:200) for 30 min followed by DyLight 649 labeled tomato lectin (Lectin) (DL-1178, Vector; 20 μg/mL) for 30 min. To confirm endothelial cell expression of CYPA and GPR75 a subset of sections labeled with CYP4A or GPR75 was incubated in CD31 (ab24590, Abcam; 1:200) for 72 h at 4°C followed by secondary incubations in Dylight 550 (ab96932, Abcam; 1:200) and then AlexaFluor 488 goat anti-mouse (A32723, Fisher; 1:200) for 30 min.

#### Identification of CYP4A and GPR75 in Pericytes

A series of parallel sections from animals that were perfused with FITC-dextran was chosen (n = 3 rats), washed in PBS and blocked. The tissue was co-incubated in CYP4A1 (RPAP-151, 1:200) or GPR75 (LSA1589; 1:200) and CD13 (ab108310, Abcam; 1:250) or CD13 (sc-13536, Santa Cruz; 1:200) for 72 h at 4°C followed by secondary incubations in Dylight (SA5-10041, Fisher; 1:200) and Dylight 550 donkey anti-goat (ab96932, Abcam; 1:200) or AlexaFluor 555 (A-21428, Fisher; 1:200) and AlexaFluor 647 (ab150075, Abcam; 1:200) for 30 min. When labeling for NG2, the same protocol was followed, but CD13 and respective secondary incubation steps were omitted and replaced by a primary incubation in NG2 (ab50009, Abcam) followed by the secondary incubation in AlexaFluor 647 donkey anti-mouse at similar concentrations, times and temperatures. Immunostaining of aSMA is described above.

#### Measurement of Capillary Diameters in CYP4A Positive Pericytes

Tissue from animals that had received an *in vivo* infusion of FITC-Dex to label the vasculature and *ex vivo* IHC labeling of CYP4A positive pericytes as described above was analyzed. Capillaries were defined as vessels less than 10 μm in diameter branching from penetrating arterioles as described by Atwell et al. (33) and Zlokovic et al. (46). Only first order and second order capillaries that were known to express aSMA in the neocortex meeting the above criteria and found to express a CYP4A positive pericyte were included in the analysis. Single plane confocal images were obtained of each capillary. The luminal diameter was measured 15 μm upstream of the CYP4A positive pericyte (measurement 1/M1) and then again at the site of the pericyte (measurement 2/M2). A total of 4 capillaries in 2 non-adjacent sections in 3 animals were measured in this manner (4 capillaries × 2 non-adjacent sections × 3 animals), *n* = 24.

#### Identification of CYP4A and GPR75 in Cultured Vascular Smooth Muscle and Pericytes

Middle cerebral arteries (MCAs) were dissected from 3 animals and pooled in order to isolate primary cerebral vascular smooth muscle cells (CVSMCs) as we have previously described (47, 48). Human brain microvascular pericytes were purchased (CAP-0030, Angio-proteomie, Boston, MA) and grown in pericyte growth media (CAP-0030). CVSMCs and pericytes were seeded on sterile glass coverslips and positioned inside cell culture plates. Fixation of cells was accomplished with 3.7% paraformaldehyde for 10 min and cells were subsequently treated with 0.1% Triton-100 for 1 h followed by blocking with 0.1% bovine serum albumin for 1 h. Cells were then co-incubated in GPR75 (LSA1589; 1:200) or GPR75 (LSA1589; 1:200) for 24 h at 4°C and then AlexaFluor 555 (A-21428, Fisher; 1:200; 30 min) at RT. Cultured pericytes were co-stained with CD13 (sc-13536, Santa Cruz; 1:200) and CYP4A1 (RPAP-151; 1:200) for 24 h at 4°C followed by AlexaFluor 555 (A32727, Fisher) and Dylight 550 (ab96932, Abcam; 1:200) for 30 min at RT.

#### Statistics

Mean values ± SEM are presented. The significance of differences in mean values between groups were determined using an unpaired Student's *t*-tests. All statistical analysis was performed using GraphPad Prism 8 (GraphPad Software, Inc., La Jolla, CA). A *P*-value < 0.05 was considered significant.

## Results

### Characterization of CYP4A and GPR75 Antibodies

We have previously validated the CYP4A1 antibody (RPAP-151) for WB using lysates from HeLa cells transfected with a CYP4A1 transposon vector as a positive control and non-transfected cells as a negative control (42). In the present study, we further validated this antibody for WB and IHC using brain tissue obtained from a CYP4A1 transgenic rat overexpression model (SS.CYP4A1) and the Dahl Salt Sensitive (SS) rat which has been documented to be deficient in the expression of CYP4A enzymes and the production of 20-HETE in the kidney and cerebral arteries relative to other strains of rats (42). Previous findings by mass spectrometry demonstrate that 20-HETE levels are decreased in cerebral vessels of the Dahl SS vs. the SS.CYP4A1 rat (42). In the present study, we confirmed by WB that the antibody RPAP-151 detects almost no CYP4A protein in whole brain homogenates isolated from the Dahl SS but detects abundant protein expression in the SS.CYP4A1 rat (Figure 2A). Liver microsomes from rats treated with fenofibrate, which massively induces the expression of CYP4A1 (49), was used as a positive control (Figure 2A). Furthermore, IHC examination of RPAP-151 labeling of whole brain tissue slices revealed abundant expression of CYP4A1 in the SS.CYP4A1 transgenic rats vs. very little expression in Dahl SS rats (Figure 2B). These new findings are consistent and build upon our previous studies regarding the specificity of the RPAP-151 antibody (42).

**Figure 1:**
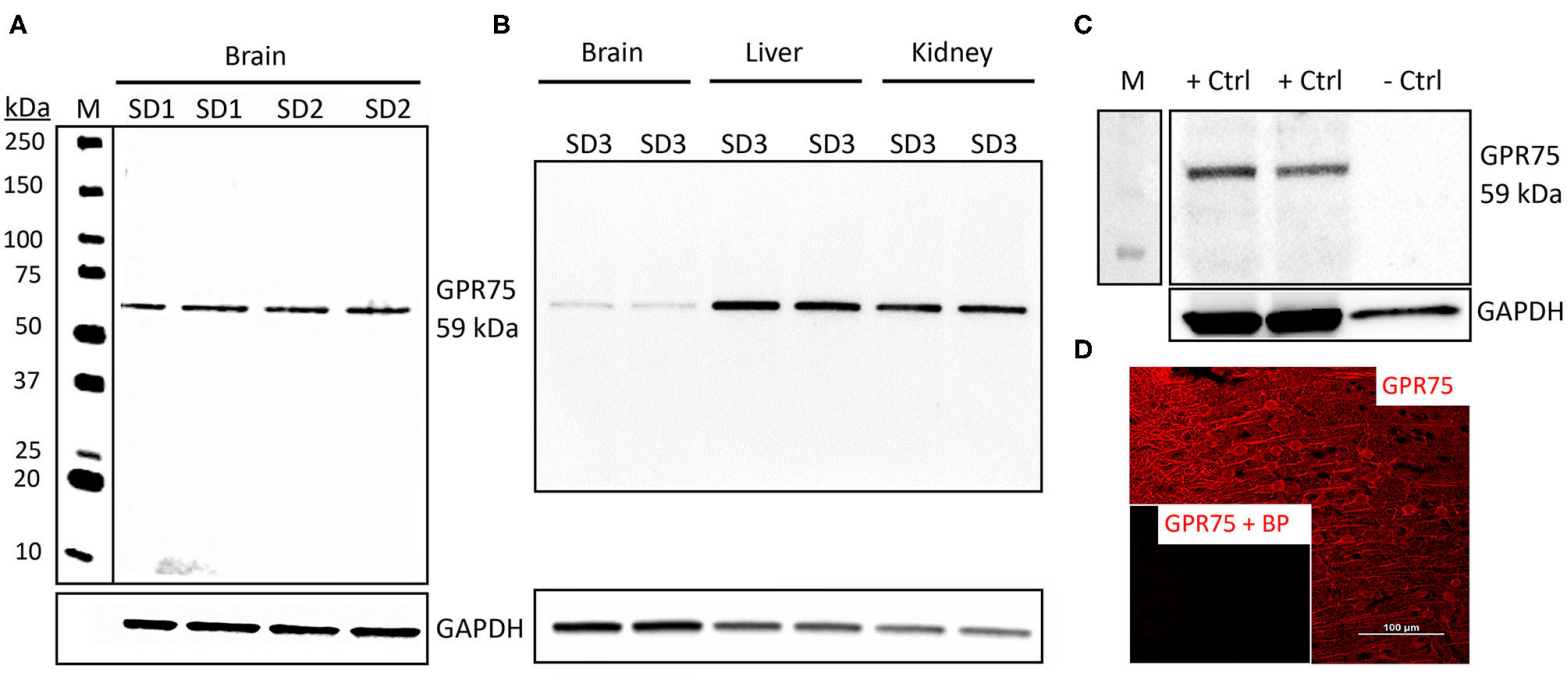
Characterization of GPR75 antibodies. **(A)** Western blot of the expression of GPR75 protein in homogenates of the brain of 2 Sprague Dawley rats (SD1 & SD2) loaded in duplicate (20 μg/mL) and probed with a GPR75 antibody (ab75581, Abcam). GAPDH was used as a loading control and lane M = molecular weight ladder. A single band at the expected molecular weight of GPR75 (59 kDa) is evident. This experiment was replicated 5 times using protein extracted from different rats. Similar results were obtained using the LS-A1589 antibody from LS-Bio. **(B)** Western blot comparing GPR75 expression in liver, kidney and brain homogenates (20 μg/mL) by ab75581 in a single SD rat (SD3) loaded in duplicate. This experiment was replicated 3 times using protein extracted from different rats. **(C)** A Western blot depicting GPR75 protein expression by ab75581 in brain homogenates (10 μg/mL) serving as a positive control (+Ctrl) vs. lysate (10 μg/mL) from HEK293 cells serving as a negative control (−Ctrl). This experiment was replicated 3 times. **(D)** Representative fluorescent immunohistochemistry image of GPR75 expression in the neocortex using the GPR75 antibody LS-A1589. Insert shows absence of labeling after pre-adsorption of the antibody with a blocking peptide (LS-E28346, LS-Bio).

The GPR75 antibody (ab75581) we used has been previously validated by Dedoni et al. using control and CRISPR-Cas9 GPR75 knockout (KO) human neuroblastoma cells. Dedoni and coworkers reported a single band in untreated cells and no band in KO neuroblastoma cells (50). In the present study, we performed Western blot experiments (*n* = 5) using ab75581 and LS-A1589 on whole brain homogenates and demonstrated that both of these antibodies identified a single specific band at the expected molecular weight of 59 kDa for GPR75 (Figure 1A) and was absent in lysate from HEK293 cells (Figure 1C). These 2 antibodies (ab75581 & LS-A1589) target different antigenic regions on GPR75. Interestingly, we also found that the expression of GPR75 protein in the brain is lower than that of the kidney and the liver by WB analysis (Figure 1B). Validation of GPR75 antibodies (LS-A1589 and LS-A1594) for IHC was accomplished by pre-adsorption with blocking peptides (LS-E28346 and LS-E28346), respectively. Pre-adsorption with blocking peptides eliminated all immunostaining (Figure 1D). The LS-A1594 produced similar results to LS-A1589 and ab75581. However, LS-A1594 labeled vascular structures slightly better than the other GPR75 antibodies. This may be explained by the different C-terminus target locations of these antibodies as seen in Table S3. A blocking peptide and the exact sequence for ab75581 is not available.

**Figure 2:**
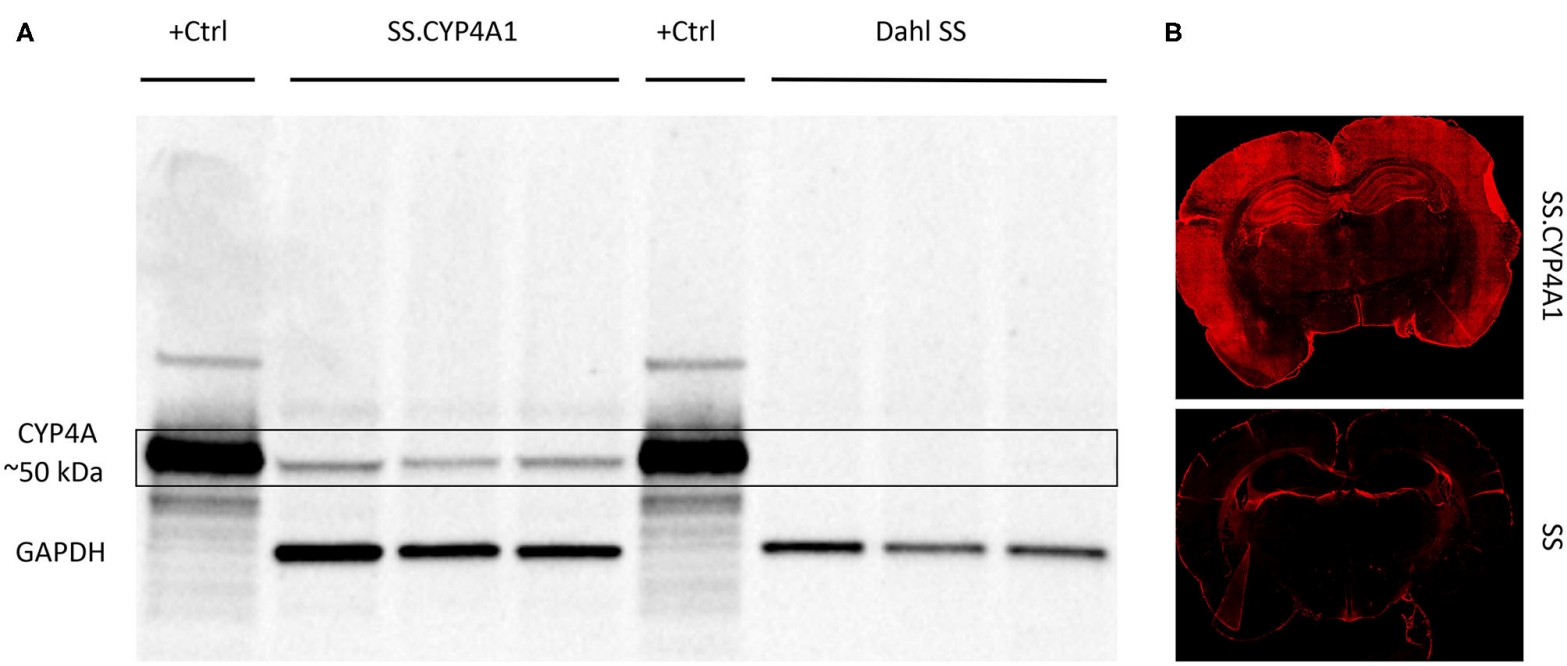
Characterization of the CYP4A1 antibody. **(A)** Western blot comparing the expression of CYP4A protein in brain homogenates (20 μg/mL) of a transgenic overexpression rat model (SS.CYP4A1) (*n* = 3) vs. the CYP4A deficient Dahl Salt Sensitive (SS) rat (*n* = 3) using a CYP4A antibody (RPAP-151, Cypex). Liver microsomes (1 μg/mL) prepared from a rat treated with fenofibrate for 3 days to induce CYP4A1 was used as positive control (+Ctrl). GAPDH was used as a loading control. A single band at the expected molecular weight of ~50 kDa is evident in the transgenic SS.CYP4A1 and was absent in the CYP4A deficient Dahl SS. This experiment was replicated 3 times. **(B)** Fluorescent immunohistochemistry images depicting abundant CYP4A expression in the brain of the SS.CYP4A1 transgenic rat relative to the Dahl SS rat by RPAP-151. This experiment was replicated in multiple sections in at least 5 rats per strain.

### Regional Expression of CYP4A and GPR75

**Figure 3:**
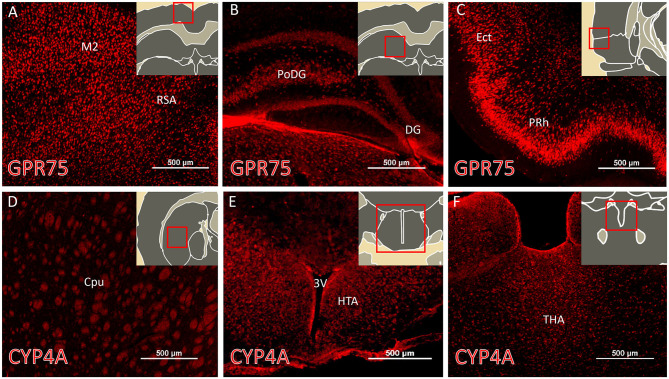
Regional expression of CYP4A and GPR75 in the forebrain. **(A–C)** Representative immunofluorescent images of GPR75 (red) expression in the brain of Sprague Dawley rats. **(D–F)** Representative immunofluorescent images of CYP4A (red) expression in the brain. (**A–F** inserts) Atlas images from the Scalable Brain Atlas based on the Waxholm Space atlas. Notable regions of abundant expression of CYP4A and GPR75 include the neocortex, entorhinal cortex (Ect), hippocampus, striatum, thalamus (THA) and hypothalamus (HTA). M2, secondary motor cortex; RSA, retrosplenial cortex; PoDG, polyphorm layer of dentate gyrus; DG, dentage gyrus; PRh, perirhinal cortex; Cpu, caudate putamen; 3V, third ventricle. Sections from 4 different animals were immunostained and imaged from at least 3 serial sections per region. Scale bar for all fluorescent images = 500 μm.

The regional expression pattern of CYP4A and GPR75 in representative brain sections (Bregma 0.20 – Bregma −5.30) are presented in Figure 3 and a summary of the areas of interest are listed in Table S2. CYP4A and GPR75 are expressed in the neocortex, entorhinal cortex, hippocampus, thalamus and hypothalamus with a relative lack of CYP4A and GPR75 expression in the brain stem.

**Figure 4:**
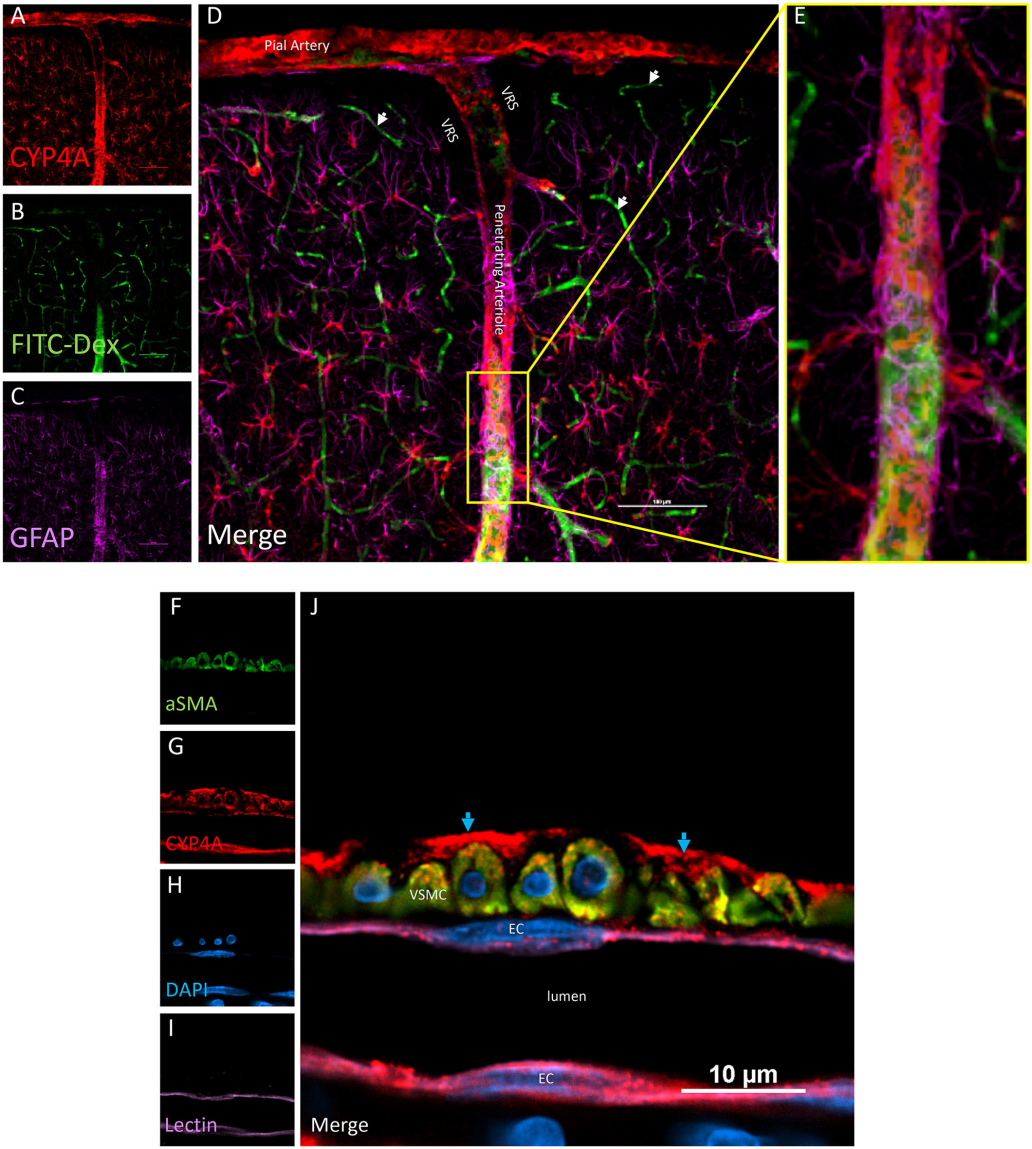
Expression of CYP4A in pial and penetrating arterioles. **(A–E)** Confocal images of a pial and penetrating arteriole in the neocortex. A penetrating arteriole is distinguished from veins by the Virchow-Robin space (VRS). CYP4A (red) is abundantly expressed in pial and penetrating arterioles labeled by *in vivo* injection of FITC-Dex (green). CYP4A is also co-expressed in astrocytes (GFAP/purple) surrounding penentrating arterioles. White arrows indicate absence of CYP4A expression on the endothelial cell layer of capillaries. Scale bar = 100 μm. **(F–J)** Single plane confocal image of a penetrating arteriole cut in cross section. Endothelial cells (EC) stained with tomato-lectin (Lectin/purple) that line the lumen co-express CYP4A (red) and yield a magenta color. Vascular smooth muscle cells (VSMC) immunostained with aSMA (green) co-express CYP4A (red) and are seen in yellow. **(J)** Red staining in the adventia (blue arrows) depicts astrocytic end feed which express CYP4A (red). Representative images were repeated in multiple vessels in 3–5 non-adjacent sections obtained from at least 3 animals.

Examination of CYP4A and GPR75 expression in various structures in the neocortex and hippocampus revealed vascular and perivascular expression along pial vessels and penetrating arterioles (Figures 4A–E). CYP4A and GPR75 expression was evident in neuronal cell bodies in the neocortex as well as the hippocampus (**Figures 6A–C**). CYP4A but not GPR75 was expressed in astrocytes (**Figures 8**, **9**). Cellular colocalization studies of CYP4A and GPR75 using specific cellular markers were used to confirm these findings and summarized in Table 1.

**Table 1:** Cellular distribution of CYP4A and GPR75.

**Cell type**	**Antibody/Stain**	**CYP4A**	**Notes**	**GPR75**	**Notes**
Neurons	NeuN	+	Expression varies by region, see Figure 7 and this table	+	Abundantly expressed, see this table
Astrocytes	GFAP	+	Expressed in subpopulations	–	No expression by IHC
Pericytes	CD13, NG2, aSMA	+	Expressed in subpopulations	+	Expression in tissue was confirmed by cell culture
Vascular smooth muscle cells	aSMA	+		+	Expression in tissue was confirmed by cell culture
Endothelial cells	CD31, Lectin	+	Not expressed on capillary endothelial cells	+	Not expressed on capillary endothelial cells

*+, expressed; −, absent*.

### Expression of CYP4A and GPR75 in Blood Vessels

**Figure 5:**
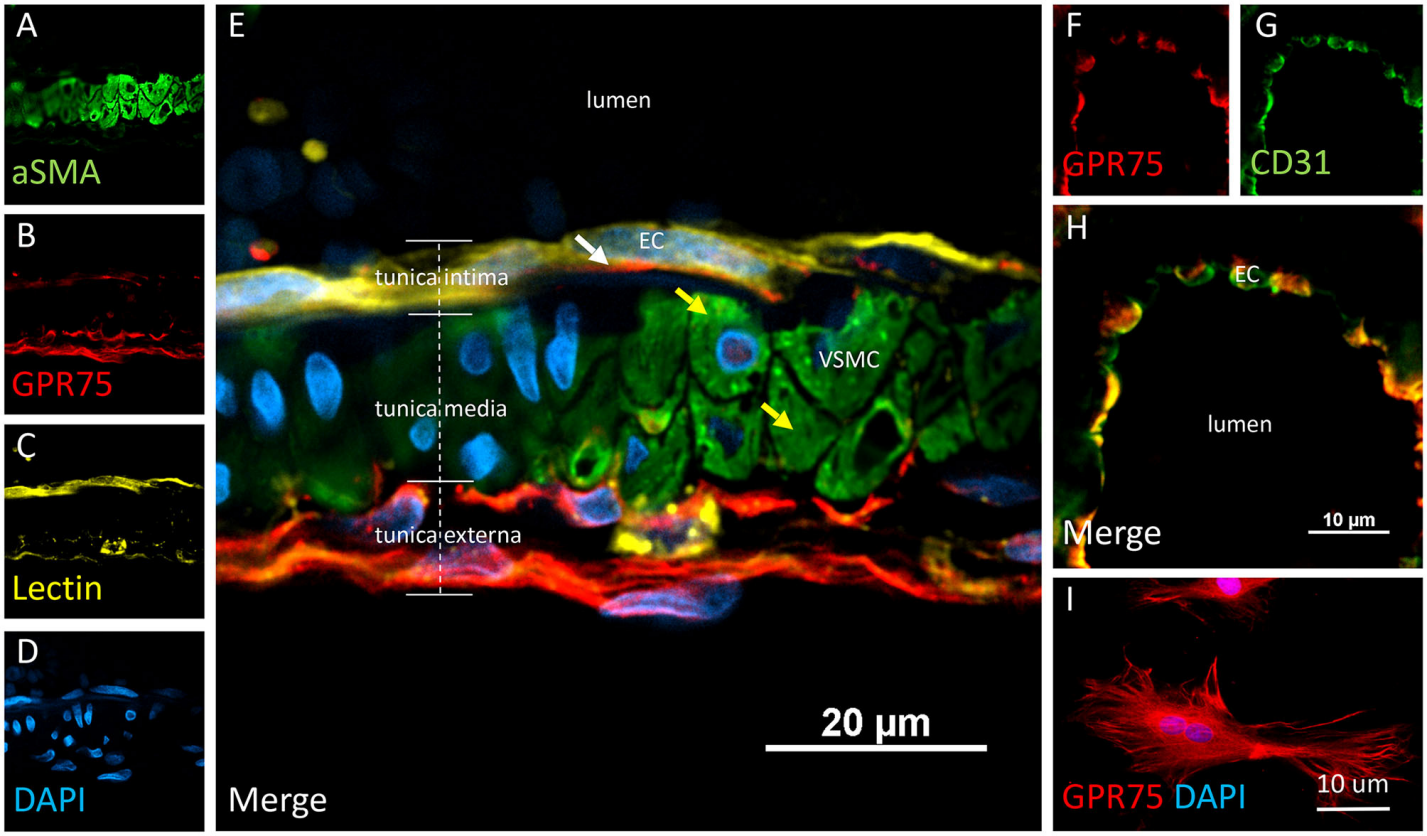
Expression of GPR75 in pial arteries. **(A–E)** Confocal image of a pial artery cut in cross section containing 2–3 layers of vascular smooth muscle cells (VSMC) immunostained by aSMA (green) in the tunica media abluminal to the endothelial cell (EC) layer of the tunica intima. The white arrow represents endothelial cells identified by tomato-lectin staining (Lectin/yellow) which co-express GPR75 (red). The yellow arrows indicate VSMCs. **(F–H)** Confocal image of a microvessel cut in cross section. Endothelial cells stained with CD31 (green) were found to co-express GPR75 (red) and are seen in yellow. GPR75 (red) is also abundantly expressed in the pial-glial basement membrane (glial limitans) in the tunica externa. **(I)** Cultured primary rat vascular smooth muscle cells stained with DAPI (blue) co-express GPR75 (red). Representative images were repeated in multiple vessels in 3–5 non-adjacent sections from at least 3 different animals. Similar results were obtained in cultured cells using 2 different GPR75 antibodies raised against different epitopes (ab75581, Abcam & LS-A1589, LS-Bio).

Co-localization experiments were performed to identify the cell types that express CYP4A and GPR75 in blood vessels. Endothelial cells in the cerebral vasculature were identified by both lectin and CD31. We found that penetration of CYP4A and GPR75 antibodies in vessels was limited most likely by the collagenous layers of the tunica externa. Longer incubation times were necessary to ensure proper penetration of antibodies and stains. Co-expression of CD31 and lectin with both CYP4A and GPR75 was observed in small cerebral vessels (Figures 4A–L, 5A–H), particularly pial and penetrating arterioles with an absence of expression in capillaries. Special attention was paid to cerebral vessels cut in cross-section to ensure that endothelial cell morphology and location inside the tunica intima was visible, providing the ability to anatomically distinguish endothelial cells from vascular smooth muscle cells. Additionally, by focusing our analysis on these vessels cut in cross-section, we could also ensure adequate penetration of antibodies into the lumen. Figures 4F–J is a cross-section of a penetrating arteriole quadruple labeled with aSMA, lectin, CYP4A, and DAPI. A single endothelial cell layer is identified by lectin and DAPI staining (Figure 4J) lining the luminal side of the vessel and found to express CYP4A. Similar co-expression patterns of GPR75 and lectin were observed in Figures 5A–E. Confirmation of endothelial cell expression of GPR75 was determined by CD31 immunolabeling (Figures 5F–H). CYP4A and GPR75 expression in the endothelial cell layer was abundantly observed in pial and penetrating arterioles yet absent in capillary endothelial cells indicated by the white arrows of Figure 4D.

VSMCs in the cerebral vasculature were immunolabeled with aSMA. Careful attention was paid to vessels cut in cross-section in order to ensure proper penetration of antibodies and distinguish VSMCs morphology and location inside the tunica media. Co-expression of aSMA and CYP4A was evident in small cerebral vessels such as pial and penetrating arterioles (Figures 4A–E) but was not found in capillaries. Some aSMA staining of CYP4A was seen in cell bodies resembling pericytes and later confirmed by NG2 and CD13 immunolabeling. Co-expression of aSMA with GPR75 was not strikingly evident in small cerebral vessels (Figures 5A–H). However, GPR75 labeling was positive in cultured rat primary VSMCs (Figure 5I).

### Expression of CYP4A and GPR75 in Neurons and Astrocytes

**Figure 6:**
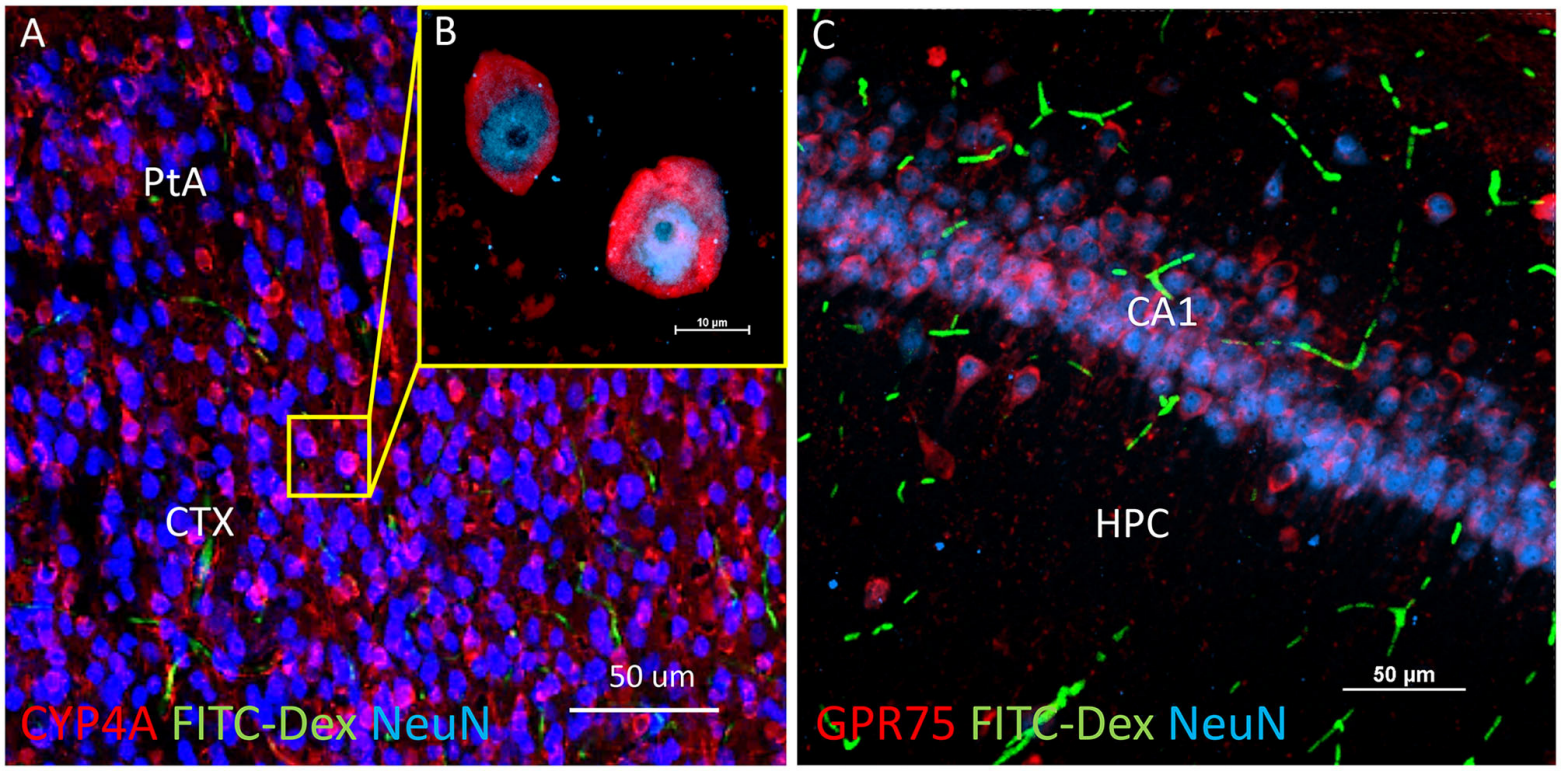
Expression of CYP4A and GPR75 in neurons of the neocortex and hippocampus. **(A)** Representative low and **(B)** high magnification confocal images of CYP4A (red) expression in cortical neurons (Neun/blue) in the parietal association area (PtA) of the neocortex (CTX). CYP4A is expressed circumferential to the nucleus which is consistent with the expected location of CYP4A in the endoplasmic reticulum. FITC-Dex (green) injected *in vivo* was used to fill and label the vasculature. **(C)** Representative confocal image of GPR75 (red) expression in neurons (NeuN/blue) of the CA1 region of the hippocampus (HPC). FITC-Dex (green) was used to fill and label the vasculature. Representative images were obtained in 3–5 non-adjacent sections from at least 3 animals.

**Figure 7:**
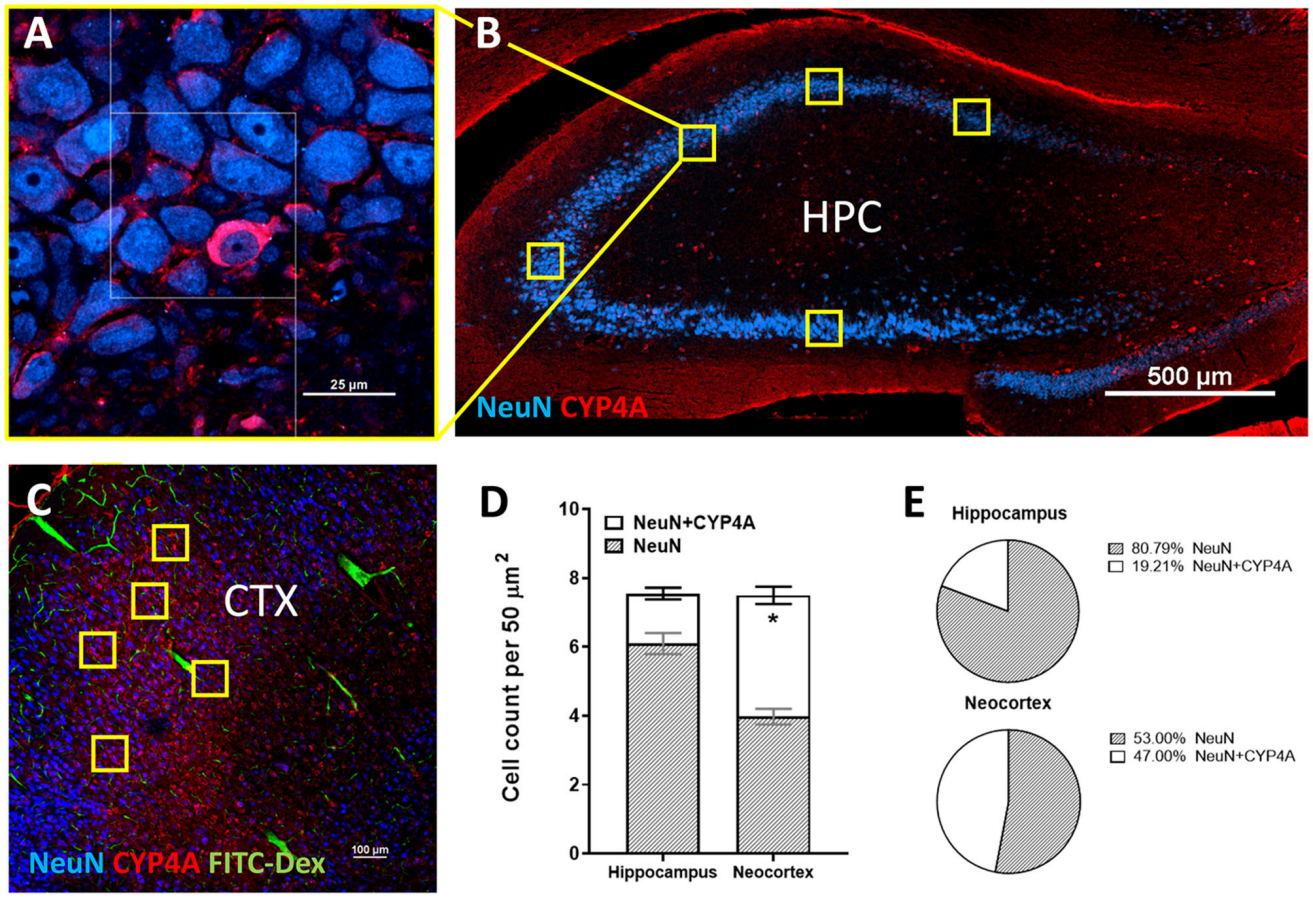
Quantification of CYP4A expression in neurons of the hippocampus and neocortex. **(A)** High and **(B)** low magnification confocal images of CYP4A (red) expression in neurons (NeuN/blue) of the hippocampus (HPC). Yellow boxes indicate the standardized measurement frames chosen to encompass CA1–CA3 regions. **(C)** Representative confocal image of CYP4A (red) expression in (NeuN/blue) neurons in the neocortex (CTX), specifically the somatosensory barrel cortex (CTX). Yellow boxes indicate standardized measurement frames. FITC-Dex (green) was injected *in vivo* to fill and label the vasculature. **(D)** Quantification of CYP4A expression in neurons (NeuN) of the hippocampus vs. neocortex. *Indicates a significant difference in the expression of CYP4A positive neurons (*P* < 0.0001) in the hippocampus vs. neocortex. All data are expressed as mean values ± SEM. **(E)** Percent of CYP4A positive neurons (NeuN) in the hippocampus and neocortex per measurement frame (50 μm^2^) (5 measurement frames per region × 4 non-adjacent sections × 3 animals), *n* = 60.

CYP4A and GPR75 was co-expressed with NeuN inside both granular and pyramidal neuron populations in all 6 layers of the neocortex as well as granular and pyramidal neurons of the hippocampus (Figures 6A–C, Figures 3A–F). The intercellular expression pattern of CYP4A was circumferential to the nucleus (Figure 6B) consistent with the location of CYP4A inside the endoplasmic reticulum (51). CYP4A expression in neurons of the hippocampus (CA1-CA3) vs. the somatosensory barrel cortex was quantified in Figures 7A–E. A significantly lower (P < 0.0001) population of CYP4A positive neurons in the hippocampus (1.45) vs. neocortex (6.10) per measurement field was observed (Figure 7D). CYP4A positive neurons represent 19% of total neurons in the hippocampus and 47% of total neurons in the neocortex per measurement field (Figure 7E).

**Figure 8:**
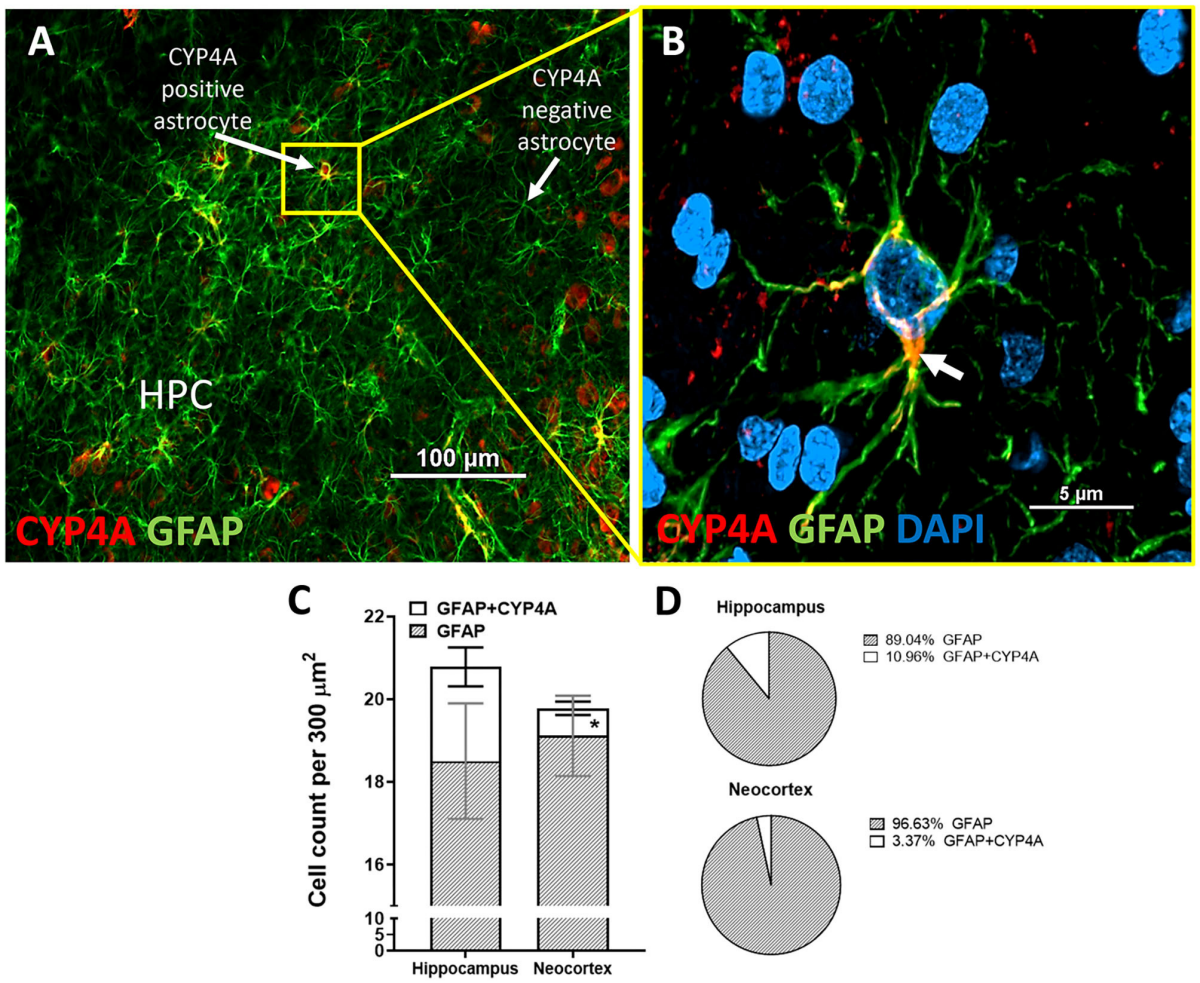
Quantification of CYP4A expression in astrocytes of the hippocampus and neocortex. **(A)** CYP4A (red) is co-expressed in a subpopulation of GFAP (green) positive astrocytes in the CA3 region of the hippocampus (HPC). **(B)** Merging of CYP4A and GFAP with DAPI (blue) reveal CYP4A expression was predominantly localized circumferential to the nucleus (white arrow) which is consistent with the location of CYP4A expression in the endoplasmic reticulum. Representative images were repeated in at least three animals in at least three different locations inside the hippocampus. **(C)** Quantification of CYP4A expression in astrocytes (GFAP) of the hippocampus vs. neocortex. *Indicates a significant difference in the expression of CYP4A positive astrocytes (*P* < 0.0001) in the hippocampus vs. neocortex. All data are expressed as mean values ± SEM. **(D)** Percent of CYP4A positive astrocytes (GFAP) in the hippocampus and neocortex per measurement frame (300 μm^2^) (3 measurement frames × 2 non-adjacent sections × 3 animals), *n* = 18.

**Figure 9:**
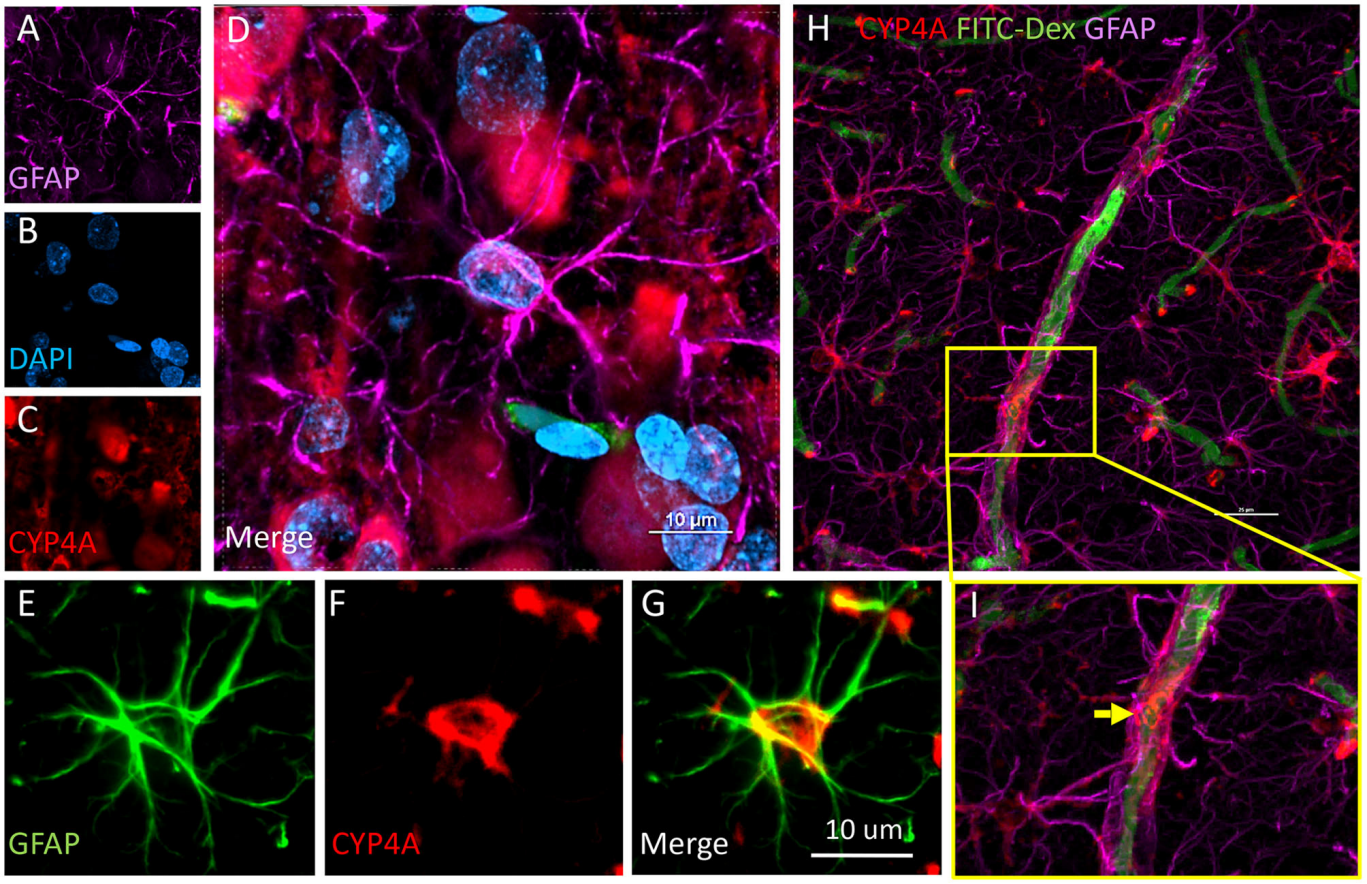
Expression of CYP4A in astrocytes and end feet surrounding microvessels. **(A–D)** Confocal images depicting CYP4A (red) expression in astrocytes (GFAP/purple). **(H,I)** Confocal images depict abundant CYP4A expression in astrocytic end feet surrounding microvessels (yellow arrow). Scale bar = 25 μm. **(E–G)** Fluorescent image depicting colocalization of GFAP (green) and CYP4A (red) in a CYP4A positive astrocyte cell body depicted in yellow. Representative images were repeated in at least 3 animals in at least 3 different locations.

CYP4A was co-expressed in GFAP positive astrocyte cell bodies in the neocortex and hippocampus (Figures 8A,B, 9A–I). The expression of CYP4A inside the cell body of the astrocyte was also found to be circumferential to the nucleus as expected inside the endoplasmic reticulum (51). Most interestingly, the co-expression of CYP4A in astrocytes was not apparent in the vast majority of astrocytes but rather in minor subpopulations (Figure 8A). A significantly lower (*P* < 0.0027) population of CYP4A positive astrocytes in the neocortex (0.67) vs. hippocampus (2.28) per measurement field was observed (Figure 8C). CYP4A positive astrocytes represent 10% of total astrocytes in the hippocampus and 3% of total astrocytes in the neocortex per measurement field (Figure 8D). In the neocortex, we also found that CYP4A was expressed inside the astrocytic end feet on penetrating arterioles and larger capillaries (Figures 4A–E, 9H,I). The astrocyte populations that co-expressed CYP4A portrayed an activated and highly branched morphology vs. less branched quiescent astrocytes that did not express CYP4A (Figure 9G).

Because receptor levels may fluctuate in response to negative or positive physiological feedback, the quantification of the CYP4A enzymes that produce 20-HETE in healthy control animals, rather than the quantification of GPR75 was desired as a baseline for future interventional studies. A regional description of both GPR75 and CYP4A was also accomplished.

### Expression of CYP4A and GPR75 in Pericytes

**Figure 10:**
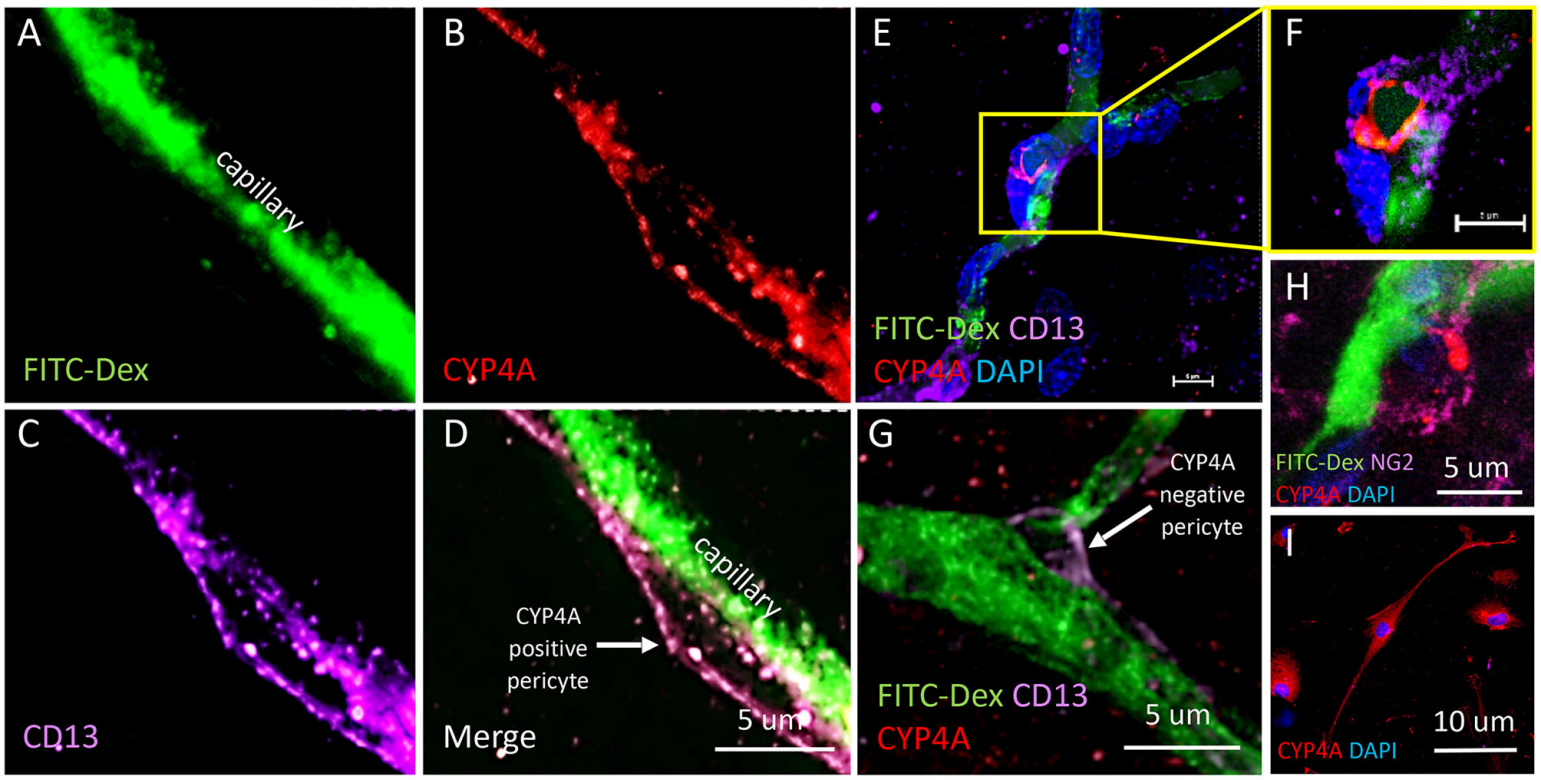
Expression of CYP4A in pericytes. **(A–D)** Representative confocal images of cerebral capillaries (FITC-Dex/green) that depict a fusiform pericyte cell body that co-expresses CYP4A (red) and CD13 (purple). **(D)** White arrow indicates a CYP4A positive pericyte. **(E)** Low magnification and **(F)** high magnification confocal images of an ovoid pericyte cell body (DAPI/blue) displaying the classic “bump on a log” morphology located on a cerebral capillary filled with FITC-Dex (green) that co-expresses CYP4A (red) and CD13 (purple). Scale bar = 5 μm. **(G)** A pericyte cell body that expresses CD13 (purple) is visible on a cerebral capillary (FITC-Dex/green). This particular pericyte does not express CYP4A (red). The white arrow indicates a CYP4A negative pericyte. **(H)** High power image of a pericyte on a capillary (FITC/Dex) that co-expresses CYP4A (red) and NG2 (purple). **(I)** Expression of CYP4A (red) was evident in cultured human cerebral pericyte cells (DAPI/blue). Representative images in tissue were repeated in at least 3 animals in at least 3 locations. Cell culture immunostaining experiments were replicated 3 times.

**Figure 11:**
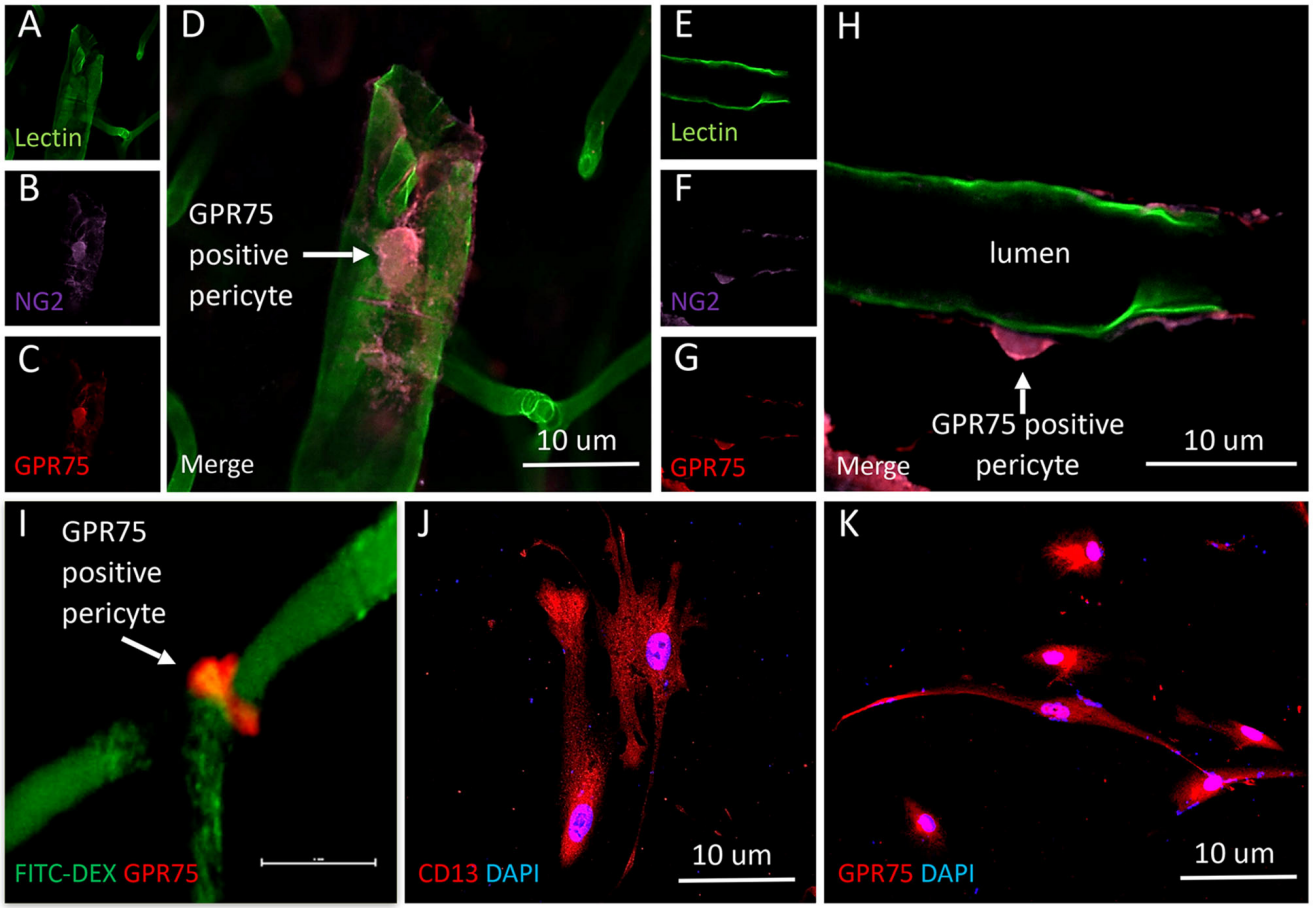
Expression of GPR75 in pericytes. **(A–H)** Confocal images depicting pericytes (NG2/purple) on pre-capillary arterioles (Lectin/green) that co-expresses GPR75 (red) yielding a magenta color. **(H)** White arrow indicates a GPR75 positive pericyte. **(I)** Confocal image of a pericyte on a capillary (FITC-Dex) that expresses GPR75 (red). **(J)** Fluorescent images of cultured human cerebral pericyte cells (DAPI/blue) that express CD13 (red), white arrow indicates a GPR75 positive pericyte, scale bar = 10 μm. **(K)** Cultured human cerebral pericytes (DAPI/blue) express GPR75 (red). Representative images in tissue were repeated in at least 3 animals in at least 3 locations. Representative images in cultured cells were completed using 2 different antibodies (ab75581, Abcam & LS-A1589, LS-Bio).

**Figure 12:**
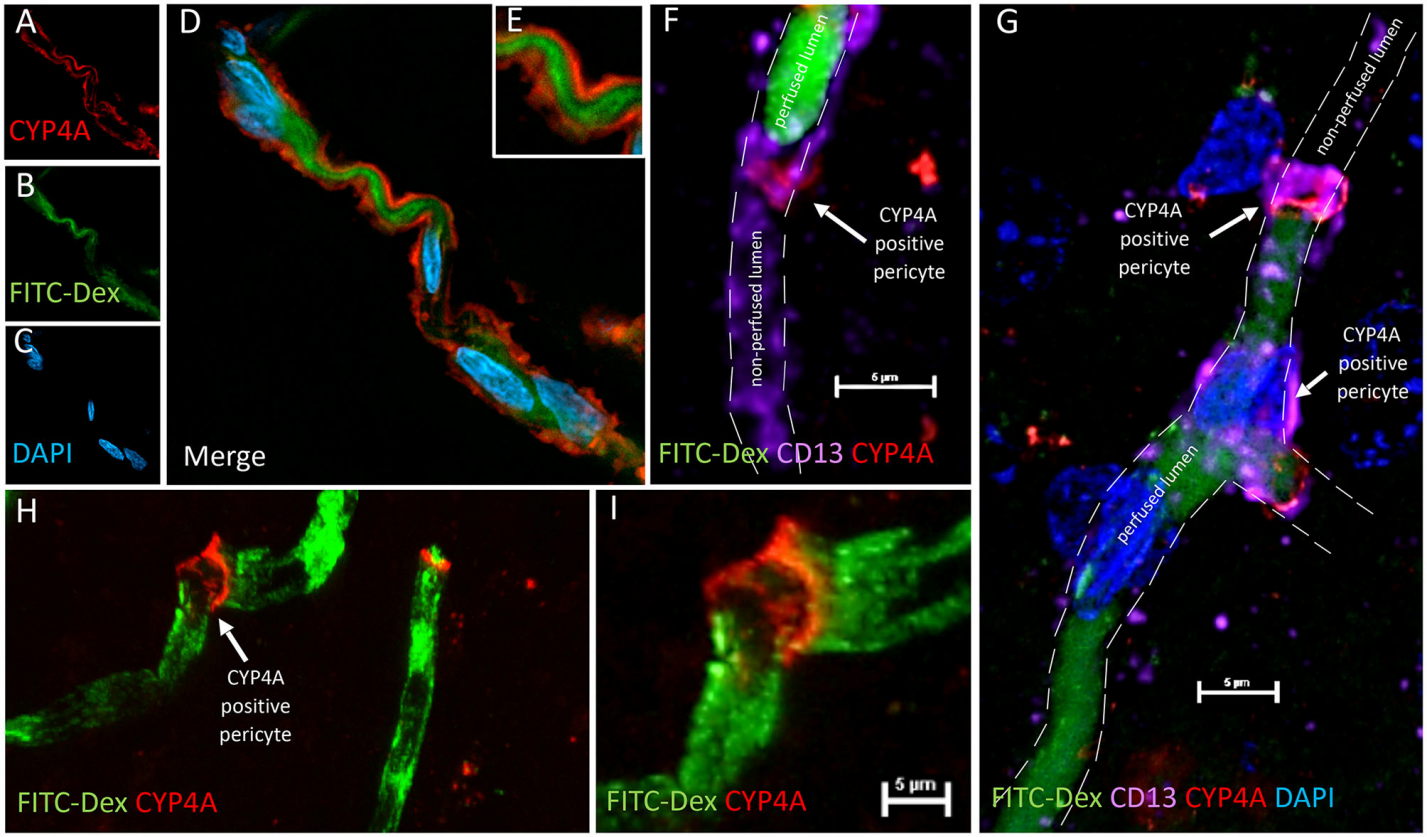
CYP4A positive pericytes are associated with bending and constriction of capillaries. **(A–E)** Confocal image of a CYP4A positive pericyte (red) expressed on a winding capillary visualized by FITC-Dex perfusion (green). **(F)** Confocal image of a CYP4A positive pericyte (red) confirmed by CD13 (purple) on a capillary visualized by FITC/Dex perfusion (green). *In vivo* perfusion of FITC-Dex (green) is blocked at the site of the pericyte. CD13 labeling of the pericyte cell body continues in the shape of the capillary lumen yet no FITC/Dex is seen downstream. White arrow indicates a CYP4A positive pericyte. Scale bar = 5 μm. **(G)** Confocal image of CYP4A positive pericytes (red) confirmed by CD13 (purple) on a capillary visualized by FITC/Dex (green). *In vivo* perfusion of FITC-Dex (green) is blocked at the site of the pericyte and not seen downstream. White arrows indicate a CYP4A positive pericyte. **(H,I)** Low and high magnification confocal image of a capillary visualized by FITC/Dex perfusion (green) that is kinked at the expression site of a CYP4A positive pericyte as indicated by the white arrow. Representative images were repeated in at least 3 animals in at least 3 locations.

The results presented in **Figures 11I**, **12H** reveal that CYP4A and GPR75 were expressed in perivascular cells along capillaries. This finding suggested that CYP4A and GPR75 may be expressed in pericytes. Immunofluorescent identification of pericytes is complicated by the fact that there is no single marker that is specific for pericytes (52). Many markers used to identify pericytes such as CD13, PDGFRβ, and NG2 are also expressed in VSMCs and glial cells (52). Therefore, it is necessary to use multiple markers and correlate location as well as morphology when identifying pericytes histologically. Cerebral arterioles and capillaries were identified by *in vivo* perfusion of FITC-Dex. Multiple CYP4A and GPR75 positive perivascular cells possessing both the characteristic fusiform or ovoid cell bodies at branch points and along capillaries (<8 μm) in diameter were visible upon high magnification (Figures 10D, 11H). CYP4A and GPR75 were found to be expressed in a subpopulation of zero, first, second, third and fourth-order pericytes. Ensheathing pericytes (Figures 11A–D) and thin strand pericytes (Figure 12D) as characterized by Shih et al. were observed to co-express CYP4A and GPR75 on pre-capillary arterioles and capillaries, respectively. Fluorescent labeling with CD13 and NG2 was used to confirm co-expression of both CYP4A and GPR75 (Figures 10A–H, 11A–H). Assessment of CYP4A and GPR75 expression in pericytes was further confirmed by immunolabeling cultured human cerebral pericytes with either CYP4A or GPR75 (Figures 10I, 11K). Both CYP4A and GPR75 were strongly expressed in these cultured pericytes and CD13 labeling of these same cells was also positive (Figure 11J). However, not all pericytes in tissue samples were found to be positive for CYP4A and/or GPR75. Many CD13 and NG2 positive pericytes were identified and were not found to express either CYP4A or GPR75 (Figure 10G).

**Figure 13:**
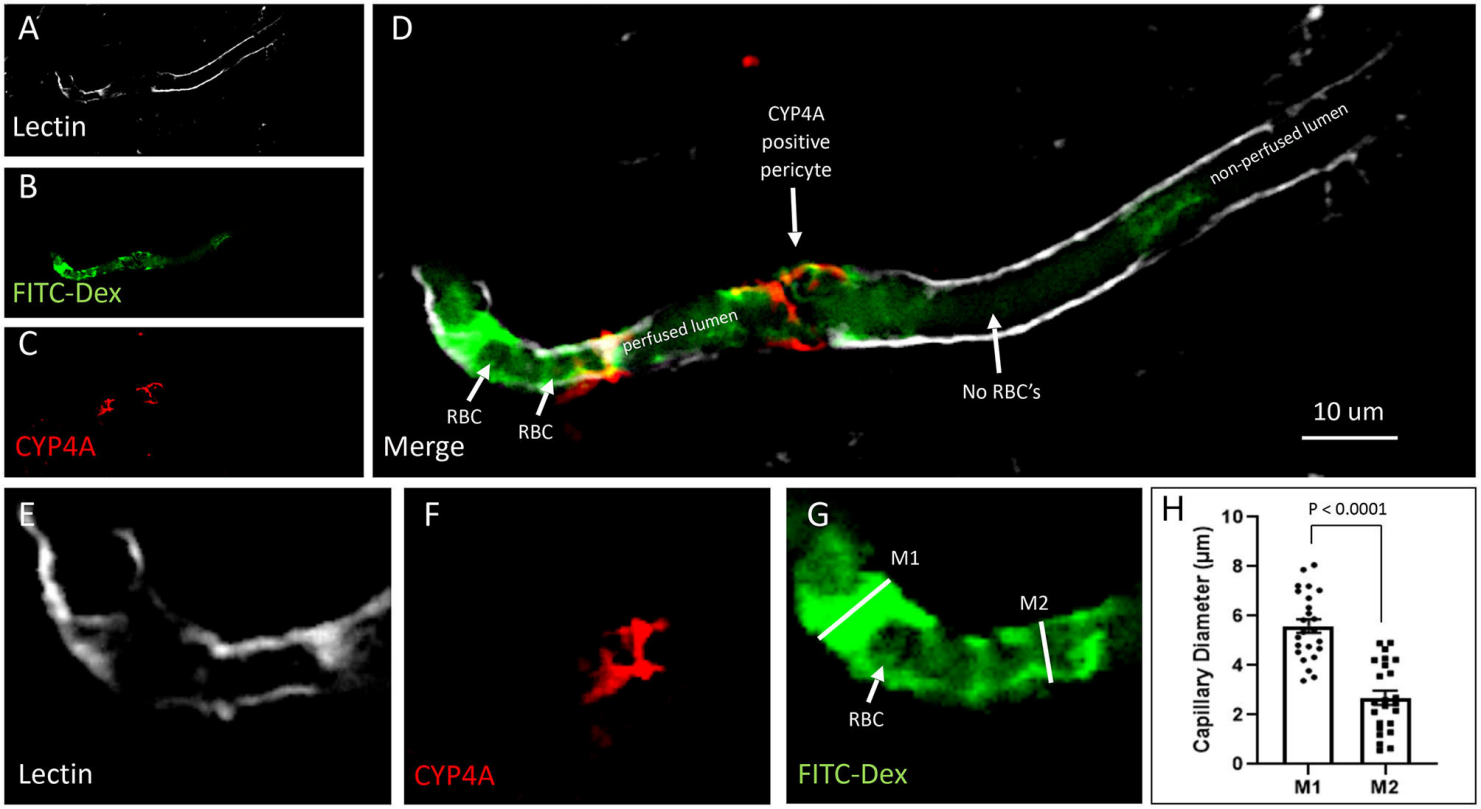
Quantification of capillary constriction associated with CYP4A positive pericytes. **(A–D)** Representative confocal images of capillaries perfused with FITC-Dex (green) *in vivo* and then labeled *ex vivo* with tomato-lectin (Lectin/white) and CYP4A (red). Examination of these vessels under high magnification reveal blockage of perfused FITC-Dex at the site of a CYP4A positive pericyte. Red blood cells (RBCs) identified upstream to the site of constriction were not seen past the point of constriction. Confirmation that the capillary was not out of plane is evident by continuation of the lumen visible by (Lectin/white) staining. White arrows indicate either the shadow of a RBC (red blood cell), absence of a RBC, or a CYP4A positive pericyte as labeled directly in the figure. **(E–G)** Capillary diameters were measure before (measurement 1 = M1) and at the site of a CYP4A positive pericyte (measurement 2 = M2). White arrow indicates a RBC. **(H)** Capillary diameters were significantly decreased (*P* < 0.0001) at the site of pericyte induced constriction. All data are expressed as mean values ± SEM (4 capillaries × 2 non-adjacent sections × 3 animals), *n* = 24.

We were surprised to see evidence of capillary constriction and blockage of red blood cell (RBC) flow when examining these CYP4A positive pericytes under high magnification (Figures 13A–H). Some capillaries that had been infused *in vivo* with FITC-Dex appeared to end (Figures 12F,G) or narrow (Figures 12A–E,H,I) at the site of a CYP4A positive pericyte. Furthermore, CD13 labeling of the pericyte cell body was observed to continue in the shape of the lumen (Figure 12F) although with no FITC-Dex illuminating the capillary distal to the CYP4A positive pericyte. To confirm that the lumen of the capillary was still patent, we stained the sections with lectin to identify the endothelial layer. We observed continuity of the lumen but attenuation of FITC-Dex flow and blockage of RBC's beyond the site of CYP4A positive pericytes (Figures 13A–D). Therefore, the lack of FITC-Dex filling of the capillary and absence of RBCs distal to the pericyte was related to occlusion of the capillary rather than the loss of the vessel.

### CYP4A Positive Pericytes Are Associated With Capillary Constriction

Capillary diameters were then measured at these sites to quantify the degree of constriction (Figure 13H). Capillary diameters were significantly decreased from 5.6 ± 0.2 to 2.7 ± 0.3 μm at the sites of CYP4A positive pericytes when compared to the diameters measured 15 μm upstream on the same capillary (Figures 13E–H). Only first and second-order pericytes were examined. The average decrease in the diameter of a vessel at the site of focal constriction (2.7 μm) is sufficient to block the flow of a typical RBCs that are 6–8 μm in diameter. Blockade of downstream perfusion with RBC's beyond the site of a CYP4A positive pericyte was observed in numerous vessels (Figure 13D). Capillary diameters were unaffected at the sites of pericytes that did not express CYP4A.

## Discussion

On a regional basis we found that CYP4A and GPR75 are highly expressed in the neocortex, entorhinal cortex, hippocampus, thalamus and hypothalamus. We also found that the expression of CYP4A was very low in the brain of Dahl SS rats relative to SD and CYP4A1 transgenic rats both by Western blot and immunostaining. These findings are consistent with previous reports that the expression of CYP4A and the formation of 20-HETE is deficient in the kidney, middle cerebral arteries and renal arterioles in Dahl SS rats when compared to other strains of rats and is restored in CYP4A transgenic Dahl SS rats.

One of the most interesting novel finding is that CYP4A is highly expressed in neurons of the hypothalamus and paraventricular nucleus that surround the 3rd ventricle. This region has long been known to play an important role in the regulation of sympathetic tone and the development of angiotensin II and DOCA salt hypertension in the spontaneously hypertensive rat (SHR) (30). This finding may provide additional information to help resolve the long-standing controversy regarding the role of CYP4A enzymes and 20-HETE in the development of hypertension. CYP4A has both pro and anti-hypertensive effects. 20-HETE inhibits sodium transport in the kidney and is thought to contribute to the development of salt-sensitive hypertension in 20-HETE deficient Dahl SS rats. On the other hand, elevation in the vascular production of 20-HETE is associated with hypertension and increased vascular tone in SHR and CYP4A/CYP4F transgenic KO models in mice. Administration of a 20-HETE inhibitor rapidly lowers blood pressure in these models. The fall in blood pressure has been interpreted to be due to inhibition of the vasoconstricting effects of 20-HETE in the vasculature. The present study suggests that perhaps 20-HETE inhibitors might lower blood pressure centrally by reducing sympathetic outflow rather than blocking 20-HETE mediated vasoconstriction.

Additionally, we found that CYP4A and GPR75 are abundantly expressed in the striatum and amygdala. This finding is consistent with previous studies indicating the susceptibility of these regions to hypoxia following transient MCA occlusion and the rescue of the ischemic core with 20-HETE inhibitors. CYP4A and GPR75 expression in the hippocampus, entorhinal cortex and corpus callosum may play a role in Alzheimer's disease and vascular dementia. Expression in these regions could have a functional significance that may help explain cognitive impairment and white matter changes observed in human studies examining CYP4A genetic variants (10) and studies in animals examining GPR75's role in neurodegeneration and amyloid-β accumulation (50).

### CYP4A and GPR75 in the Neurovascular Unit

A major novel finding of the present study was the identification of CYP4A and GPR75 in the components of the NVU, including endothelial cells and VSMCs in pial and penetrating arterioles, the glial limitans, capillary pericytes and perivascular astrocytic endfeet. CBF, the permeability of the BBB, neurovascular coupling, angiogenesis, neurogenesis, neurotransmitter production and turnover, as well as interactions of the extracellular matrix are all regulated by the NVU (53). 20-HETE is a potent vasoconstrictor that plays a positive role in the myogenic response and autoregulation of CBF (42), BBB integrity (42, 54) and is pro-angiogenic (55). However, 20-HETE has also been implicated to have a pathologic role in endothelial dysfunction, oxidative stress and metabolic syndrome (56). The location of both the enzymes that produce 20-HETE and its receptor inside the NVU reveals new light on the possible mechanisms at play that may explain 20-HETE's role in impaired autoregulation of CBF, ischemic stroke, hemorrhagic stroke and vascular dementia.

### 20-HETE and Cerebral Blood Flow

The expression of CYP4A and GPR75 in endothelial cells, vascular smooth muscle cells and pericytes is consistent with a role for 20-HETE in the regulation of cerebrovascular tone. Our results indicate that 20-HETE, produced by either endothelial cells, VSMCs, astrocytes or pericytes in the cerebrovascular system may act directly on 20-HETE receptors located on VSCMs or alpha-smooth muscle expressing pericytes to mediate vasoconstriction.

Although the constrictive properties of capillary pericytes is still very much a topic of debate, recent *in vivo* studies using 2 photon microscopy provide evidence that capillary pericytes can constrict and influence the local distribution of capillary perfusion (33, 57). Much of the criticism regarding the constrictive nature of pericytes has centered around the lack of machinery, specifically the expression of aSMA in capillary pericytes, as evident by immunohistology studies. However, Alarcon-Matinez et al. recently reported that the failure to detect aSMA in pericytes might be due to the rapid depolymerization of filamentous actin in pericytes during typical fixation protocols and demonstrated that the expression of aSMA could be preserved with novel fixation methods (58). We now report that the CYP4A, which produces 20-HETE, previously reported to influence capillary diameter (33), is expressed in capillary pericytes and that these pericytes also express the putative 20-HETE receptor, GPR75. Moreover, we report that CYP4A positive pericytes are associated with a focal decrease in the diameter of capillaries and may restrict the downstream flow of plasma and RBCs. Future studies should be undertaken to assess whether blocking 20-HETE or it's receptor can reverse this phenomenon and restore flow.

Targeting pericytes in cerebrovascular disease is an emerging area of research (59). In disease states such as ischemic stroke, an abundant release of arachidonic acid, the substrate for 20-HETE production, is observed due to cell death. This increase of substrate in the presence of the CYP4A enzyme would be expected to increase 20-HETE production and provide the opportunity for 20-HETE to act on neighboring pericytes. This potential mechanism may help explain why pericytes die in a state of constricted rigor (33). These findings are consistent with reports that inhibitors of 20-HETE reduce infarct size in ischemic stroke and improve the no-reflow phenomena following ischemic stroke.

Pericyte loss and impairment has also been implicated in Alzheimer's disease and vascular dementia (57). This study elucidates the location of a G-protein coupled receptor, linked to 20-HETE and cerebrovascular pathology, on capillary pericytes. As such, future studies of the therapeutic value of GPR75 antagonists on disease states such as ischemic stroke, Alzheimer's disease and vascular dementia should be explored.

### 20-HETE and the BBB

The location of CYP4A and GPR75 in the endothelial layer of small cerebral vessels has implications into the possible role of 20-HETE in the permeability and integrity of the BBB. Recent studies using a 20-HETE synthesis inhibitor (HET0016) demonstrated that the permeability of the BBB and brain edema after traumatic brain injury was reduced by HET0016 (54). Lu et al. proposed that the mechanisms may be due to the attenuation of oxidative stress and the increase of superoxide dismutase and total antioxidant capacity which regulate expression patterns of tight junction proteins and MMP-9 (54). Conversely, 20-HETE may play a protective role in BBB integrity. Fan et al. demonstrated that the Dahl SS rat is deficient in the vascular formation of 20-HETE and exhibits BBB leakage following transient elevations in blood pressure and that BBB integrity improved in CYP4A1 transgenic SS rats (42). The protective role of 20-HETE in BBB leakage may be explained by the present study where we report abundant CYP4A expression in the pial and penetrating arterioles. Autoregulation of blood flow through the myogenic response in the pial and penetrating arteriole may better protect downstream capillary beds from elevations in blood pressure.

The identification of a receptor on the vascular endothelium is an ideal target for future therapeutics such as GPR75 antagonists or agonists. Additionally, antagonism of GPR75 by a small molecule or peptide that does not have to cross the BBB possesses an advantage over fat-soluble 20-HETE antagonists, which readily cross the BBB and may cause off target effects.

### 20-HETE and Neurodegeneration

We also found that CYP4A and GPR75 are abundantly expressed in neurons throughout the forebrain. Ignatov et al. have reported a neuroprotective effect from amyloid-β accumulation by GPR75 activation via the endogenous chemokine RANTES/CCL5. Yet, 20-HETE has consistently been shown to be neurotoxic and blocking 20-HETE is neuroprotective in both human and animal studies (29, 60–65). Whether or not CCL5 is another endogenous ligand of GPR75 remains questionable as β-arrestin assays designed to confirm pairing have failed (66). Although, the interaction between 20-HETE and CCL5 cannot be ruled out. CCL5 may act as a competitive antagonist of 20-HETE, and one can speculate that the neuroprotective effect of CCL5 on GPR75 might be attributed to blockade of oxidative stress and the neurotoxic effects of 20-HETE. Therefore, it will be prudent to examine GPR75 antagonists as potential neuroprotective therapies for Alzheimer's disease and cerebrovascular disease.

### 20-HETE and Astrocytic Regulation of Functional Hyperemia

Astrocytes of the NVU affect neurovascular coupling and modulate CBF. Identification of CYP4A in astrocytic cell bodies and perivascular end feet has profound implications on functional hyperemia. Astrocytes are a heterogeneous group of glial cells, and Sosunov et al. pointed out that astrocytes differ in morphology, intermediate fibers, protein expression, and function (67). Our findings indicate that not all astrocytes express CYP4A but rather it is only expressed in a subpopulation of astrocytes. This suggests that astrocytes may upregulate CYP4A based on physiological demand. Using brain slices, Atwell et al. reported that *in vivo* blockade of 20-HETE synthesis by nitric oxide was necessary to attenuate vasoconstriction during functional hyperemia. Atwell et al. hypothesized that either astrocytes or neurons may be the source of 20-HETE production leading to constriction of capillaries and penetrating arterioles (33).

### 20-HETE and Astrogliosis

Our results suggest that the subpopulation of astrocytes that express CYP4A possess a reactive morphology in contrast to the largely quiescent phenotype in CYP4A negative astrocytes. These results are consistent with astrocytic production of 20-HETE reported in cell culture (68). Additionally, astrogliosis in the hippocampus caused by arterial hypertension, ischemic stroke, and Alzheimer's disease may lead to pathologic increases in 20-HETE levels exacerbating hypoxic states, inducing ROS and thus prolonging recovery. Targeting astrocytes in Alzheimer's disease and cerebrovascular disorders is becoming an emerging topic of interest (69, 70). Therefore, inhibition of astrocyte-derived 20-HETE production or blockade of GPR75 on VSMCs and pericytes may be an attractive therapeutic target for future intervention.

### Isoforms of GPR75 and/or Additional 20-HETE Receptors

It is reasonable to suspect that there may be different isoforms of GPR75 or other 20-HETE receptors expressed in different tissues. In this regard, Garcia et al. reported that GPR75 was expressed in human vascular endothelial cells and increased the expression of angiotensinogen by activating the MAPK system (34). However, they were unable to demonstrate the expression of GPR75 in renal or cerebral vascular smooth muscle cells which contract to 20-HETE via activation of the PLC/IP3 pathway. Nor did they find expression of GPR75 in the proximal tubule or thick ascending loop of Henle in the kidney in which 20-HETE inhibits sodium transport through the IP3 pathway (18). This suggests that there may be multiple isoforms or splice variants of GPR75 expressed in different tissues or that GPR75 is not the only receptor for 20-HETE. Indeed, a recent study reported that 20-HETE promotes insulin secretion in the pancreas by activating a free fatty acid receptor (FFAR1/GPR40) (71).

## Conclusion

The present study localized 20-HETE producing enzymes (CYP4A) and the newly identified 20-HETE receptor (GPR75) to cells of the NVU and regions of the forebrain implicated in cerebrovascular pathology. Most surprisingly, CYP4A positive pericytes were associated with capillary constriction and occlusion of the flow of plasma and RBCs. 20-HETE has consistently been implicated in human cerebrovascular disease. The specific cellular locations inside the NVU may now help explain mechanisms responsible for 20-HETE associated pathologies and highlight GPR75 as a druggable target for future interventional studies.

## Data Availability Statement

The raw data supporting the conclusions of this article will be made available by the authors, without undue reservation.

## Ethics Statement

The animal study was reviewed and approved by Institutional Animal Care and Use Committee (IACUC) of the University of Mississippi Medical Center.

## Author Contributions

EG-F, RR, FF, and LC conceived and designed experiments. EG-F, DS, KL, BN, YL, and ML performed the experiments. EG-F and KL: cell counting. EG-F, RR, FF, and LC: analysis of data. EG-F and RR wrote the paper. CW: assistance with clinical significance. All authors contributed to the article and approved the submitted version.

## Conflict of Interest

The authors declare that the research was conducted in the absence of any commercial or financial relationships that could be construed as a potential conflict of interest.
