# Supplementary material for: 20-HETE Enzymes and Receptors in the Neurovascular Unit: Implications in Cerebrovascular Disease
Source: Front Neurol. 2020 Sep 4;11:983. doi: 10.3389/fneur.2020.00983 (PMC7499024; doi:10.3389/fneur.2020.00983)
Supplement: Supplementary file 1 [file Data_Sheet_1.PDF]

Table S1 Primary CYP4A or GPR75 antibodies used in this study

Name of Antibody	Location or amino acid sequence of immunogen if known	Manufacturer, Catalog No., Type	Concentration and times for IHC and WB incubations	Notes
CYP4A1	4A1 region of C-terminus	Cypex, R-PAP 151, Goat polyclonal to Rat CYP4A1	1:1,000 ABC-TSA IHC method for 17h at RT or 1:200 for 72h at 4°C, 1:500 for WB	known to react with CYP4A1, 4A2 and 4A3, IHC results similar to ab3573
CYP4A	DPSRFAPDSPRHSHS	Abcam, ab3573, Rabbit polyclonal to CYP4A	1:1,000 ABC-TSA IHC method for 17h at RT or 1:200 for 72h at 4°C, 1:500 for WB	known to react with CYP4A2, A10, A12 and A14, IHC results similar to RPAP-151
GPR75	450-500* region of C-terminus	LS-Bio, LS-A1589, Rabbit polyclonal to Human GPR75,	1:4,000 ABC-TSA method at RT or 1:200 for 72h at 4°C, 1:1,000 for WB	IHC results similar to ab75581
GPR75	400-450* region of C-terminus	LS-Bio, LS-A1594, Rabbit polyclonal to Human GPR75	1:4,000 ABC-TSA method at RT or 1:200 for 72h at 4°C, 1:1,000 for WB	better identification of vascular cell types and pericytes by IHC
GPR75	380-430* region of C-terminus	Abcam, ab75581, Rabbit polyclonal to GPR75	1:4,000 ABC-TSA method at RT or 1:200 for 72 h at 4°C, 1:1,000 for WB	IHC results similar to LS-A1589 & ab75581

***Footnote: GPR75 Amino Acid Sequence**

1 MNTSAPLQNVPNATLLNMPPLHGGNSTSLQEGLRDFIHTATLVTCTFLLAIIFCLGSYGN
70 FIVFLSFFDPSFRKFRTNFDFMILNLSFCDLFICGVTAPMFTFVLFFSSASSIPDSFCFT
130 FHLTSSGFVIMSLKMVAVIALHRLRMVMGKQPNCTASFSCILLLTLLLWATSFTLATLAT
190 LRTNKSHLCLPMSSLMDGEGKAILSLYVVDFTFCVAVVSVSYIMIAQTLRKNAQVKKCPP
250 VITVDASRPQPFMGASVKGNGDPIQCTMPALYRNQNYNKLQHSQTHGYTKNINQMPIPSA
310 SRLQLVSAINFSTAKDSKAVVTCVVIVLSVLVCCLPLGISLVQMVLSDNGSFILYQFELF
370 GFTLIFFKSGLNPFIYSRNSAGLRRKVLWCLRYTGLGFLCCKQKTRLRAMGKGNLEINRN
430 KSSHHETNSAYMLSPKPQRKFVDQACGPSHSKESAASPKVSAGHQPCGQSSSTPINTRIE
490 PYYSIYNSSPSQQESGPANLPPVNSFGFASSYIAMHYYTTNDLMQEYDSTSAKQIPIPSV

Table S2 Regional expression of CYP4A and GPR75

Name of brain area	Density of CYP4A cell bodies	Density of GPR75 cell bodies
neocortex	+++	+++
corpus collosum	+	+
hippocampus CA1	+	+++
hippocampus CA2	+	++
hippocampus CA3	+	+
hippocampus dentate gyrus	+++	+++
habenular nuclei	++	++
amygdala	+	++
thalamus	+++	+++
brain stem	-	-
periaqueductal grey	+	+
basal forebrain	++	++
hypothalamus	++	++
striatum	+	+
entorhinal cortex	+++	+++
piriform cortex	++	+++
glial limitans	++	++
paraventricular nucleus	++	++

[1]**Footnote: +++ abundant; ++ moderate; + sparse; - absent/nearly absent**
